# Cell adhesion in plants is under the control of putative *O*-fucosyltransferases

**DOI:** 10.1242/dev.132308

**Published:** 2016-07-15

**Authors:** Stéphane Verger, Salem Chabout, Emilie Gineau, Grégory Mouille

**Affiliations:** 1 Institut Jean-Pierre Bourgin, INRA, AgroParisTech, CNRS, Université Paris-Saclay, RD10, 78026 Versailles Cedex, France; 2 Université Paris-Sud, Université Paris-Saclay, 91405 Orsay Cedex, France

**Keywords:** Cell adhesion, *O*-fucosyltransferases, Cell wall integrity, *Arabidopsis thaliana*

## Abstract

Cell-to-cell adhesion in plants is mediated by the cell wall and the presence of a pectin-rich middle lamella. However, we know very little about how the plant actually controls and maintains cell adhesion during growth and development and how it deals with the dynamic cell wall remodeling that takes place. Here we investigate the molecular mechanisms that control cell adhesion in plants. We carried out a genetic suppressor screen and a genetic analysis of cell adhesion-defective *Arabidopsis thaliana* mutants. We identified a genetic suppressor of a cell adhesion defect affecting a putative *O*-fucosyltransferase. Furthermore, we show that the state of cell adhesion is not directly linked with pectin content in the cell wall but instead is associated with altered pectin-related signaling. Our results suggest that cell adhesion is under the control of a feedback signal from the state of the pectin in the cell wall. Such a mechanism could be necessary for the control and maintenance of cell adhesion during growth and development.

## Abstract (precis)

**Highlighted article:** Pectin-related signaling, rather than absolute pectin levels, are proposed to contribute to cell adhesion during growth and development in *Arabidopsis thaliana.*

## INTRODUCTION

Cell-to-cell adhesion in plants is established during cell division by the formation of a new cell wall between two daughter cells (Jarvis et al., 2003). The plant then has to contend with the fact that the large majority of its cells are fixed and will retain the same neighbor cells throughout their life. However, the cell wall is a very dynamic compartment. Its constant synthesis and remodeling mediate the growth and development of the plant, and feedback signals concerning the integrity of the cell wall provide vital cues for the plant (Wolf et al., 2012a).

A deficiency in pectin synthesis was previously shown to lead to a loss of cell adhesion (Bouton et al., 2002; Mouille et al., 2007). Mutations in *QUASIMODO1* (*GAUT8*) and *QUASIMODO2* (*TSD2*, *OSU1*), which respectively encode a putative galacturonosyltransferase of the GT8 family of glycosyltransferases (www.cazy.org) and a putative pectin methyltransferase, lead to a 50% reduction in homogalacturonan (HG; the main component of the pectins) content, and a clear cell-detachment phenotype (Bouton et al., 2002; Mouille et al., 2007). Another cell adhesion-defective mutant is affected in FRIABLE1 (FRB1), a putative *O*-fucosyltransferase (Neumetzler et al., 2012). However, contrary to the *quasimodo* mutants, *frb1* does not show a decrease in HG content in the cell wall but shows various cell wall modifications (Neumetzler et al., 2012). In addition, other *Arabidopsis* mutants have been shown to be defective in HG content in the cell wall at comparable levels to *quasimodo* mutants, such as *irregular xylem 8* (Persson et al., 2007), *pectin methylesterase 3* (Guénin et al., 2011), the overexpressor of *POLYGALACTURONASE INVOLVED IN EXPANSION 1* (Xiao et al., 2014) and the *cotton golgi-related 2*/*3* double mutant (Kim et al., 2015), but were not reported to display cell adhesion defects. Thus, the link between pectin content and cell adhesion, as well as the actual causes of cell adhesion defects in the mutants mentioned above, remain obscure.

## RESULTS AND DISCUSSION

### A cell adhesion defect suppressor screen

**Fig. 1.**
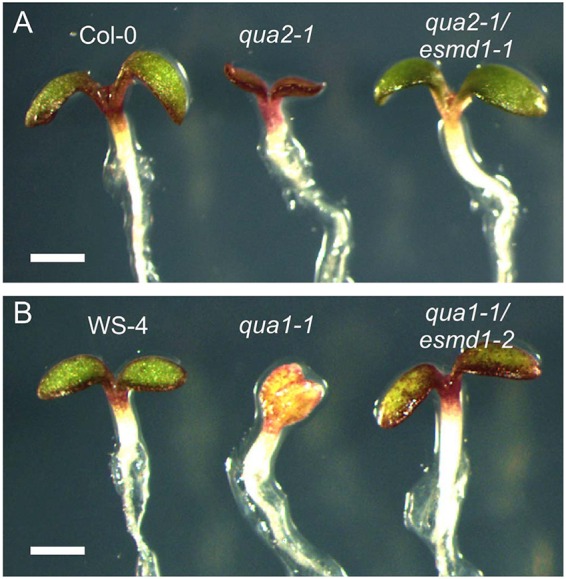
***qua1* and *qua2* primary suppressor screen.** Phenotypes of (A) Col-0, *qua2-1*, *qua2-1/esmd1-1* and (B) Ws-4, *qua1-1* and *qua1-1/esmd1-2* light-grown *A. thaliana* seedlings on a culture medium supplemented with 3% of sucrose. Among other isolated suppressor lines, the *esmd1-1* mutant was isolated as a suppressor of *qua2-1* (Col-0) and *esmd1-2* as a suppressor of *qua1-1* (Ws-4). These growth conditions were used for an efficient primary screening owing to the high sensitivity of the *qua1* and *qua2* mutants to high sucrose concentrations. Scale bars: 1 mm.

In order to identify new molecular players involved in cell adhesion, a genetic suppressor screen was carried out on EMS mutagenized *qua1-1* and *qua2-1* lines. These lines were previously shown to have a clear cell-detachment phenotype (Bouton et al., 2002; Mouille et al., 2007) and to be very sensitive to carbon/nitrogen imbalance as revealed by an accumulation of anthocyanin and substantially impaired growth and development (Fig. 1) (Bouton et al., 2002; Gao et al., 2008; Krupková et al., 2007). As a primary screen, M2 seeds of both lines were grown on a high sucrose (3%) medium and suppressor lines were screened for restored growth and greening (Fig. 1). We then checked for restored cell adhesion among the different suppressor lines, consistency of the phenotype at the M3 generation, and the presence of a single recessive suppressor locus. We thereby isolated a set of mutants affecting different loci. In reference to Victor Hugo's novel *Notre-Dame de Paris*, we named the genes affected by the second site mutations as *ESMERALDA* (*ESMD*), since their mutated form suppresses the effect of a mutation in the *QUASIMODO* genes.

### ESMERALDA1 is a widely expressed Golgi-localized putative *O*-fucosyltransferase

From our set of suppressor lines, a combination of genetic mapping and whole-genome sequencing allowed us to identify two independent alleles of At2g01480 (Figs S1 and S2). The *esmd1-1* mutant was isolated as a suppressor of *qua2-1* (Col-0), and *esmd1-2* as a suppressor of *qua1-1* (Ws-4) (Fig. 1). *ESMERALDA1* (*ESMD1*) represents a novel locus encoding a putative *O*-fucosyltransferase. It belongs to a group of 39 *Arabidopsis* proteins that possess a transmembrane domain and a predicted *O*-fucosyltransferase domain (Pfam ID number PF10250) related to the GT65 family of glycosyltransferases (Hansen et al., 2009, 2012; Neumetzler et al., 2012; Wang et al., 2013). *In vivo* observation of a GFP-tagged version of the ESMD1 protein, using a *p35S::ESMD1:GFP* construct transiently expressed in *N. benthamiana*, as well as colocalization experiments with a Golgi marker, revealed Golgi localization of the protein (Fig. S3, Movie S1). A GUS construct harboring the 2168 bp upstream promoter region of *ESMD1* was then used to study the gene expression pattern. In our growth conditions, *ESMD1* (GUS) expression is present throughout the seedling (Fig. S3).

ESMD1, along with the other putative *O*-fucosyltransferases of plants, possess conserved motifs characteristic of the superfamily of fucosyltransferases (Hansen et al., 2009) as well as the GDP-fucose protein *O*-fucosyltransferase signature (IPR019378; Fig. S2), which indicates that they should act as fucosyltransferases of proteins containing Epidermal growth factor (EGF)-like repeats (Wang et al., 2001) or Thrombospondin type 1 repeats (TSRs) (Luo et al., 2006). Although we were not able to find *Arabidopsis* proteins containing TSR domains, we identified a number of proteins containing EGF-like domains. However, only a subset of these contain the conserved C2-X(3-5)-S/T-C3 site that is the described substrate of *O*-fucosyltransferases (see Fig. 3C, Table S1) (Takeuchi and Haltiwanger, 2014). Interestingly, the list of putative protein substrates contains mostly receptor-like kinases, suggesting a role in signaling. However, with this analysis we cannot exclude the possibility that the plant putative *O*-fucosyltransferases have other types of substrates and activities and, unfortunately, we have been unable to characterize the enzymatic activity of ESMD1 or identify its substrates. Future work will focus on these aspects.

### Mutations in putative *O*-fucosyltransferases affect the state of cell adhesion in plants

**Fig. 2.**
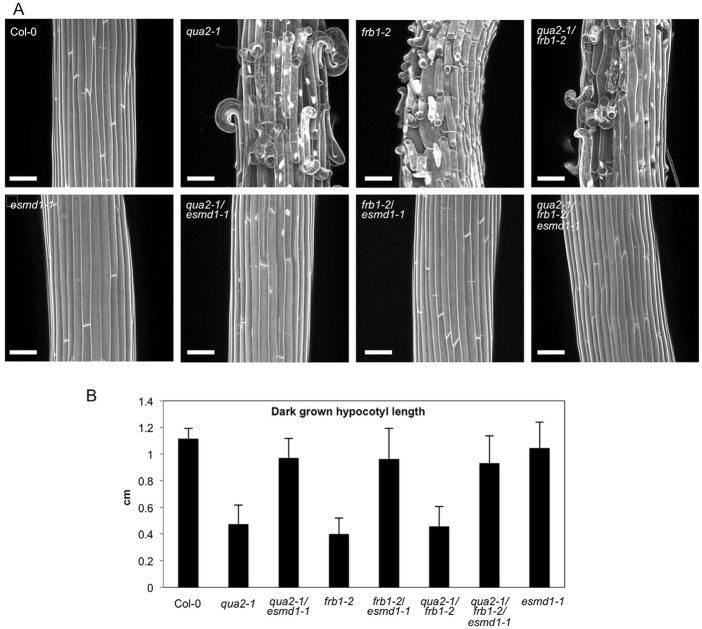
***qua2*, *frb1* and *esmd1* affect cell adhesion in the same pathway.** (A) *z*-projections of confocal stacks from representative, propidium iodide-stained, 4-day-old dark-grown hypocotyls, revealing the state of cell adhesion in the different mutant lines. (B) Length of 4-day-old dark-grown hypocotyls of the mutant lines, showing the effect of loss of cell adhesion on hypocotyl elongation. Average value with standard deviation, of three biological replicates of 20 seedlings each. Scale bars: 75 μm.

Interestingly, the *esmd1* single mutant does not seem to show any phenotype when compared with the wild type (Fig. 2, Fig. S3). Surprisingly, a mutant in *FRB1* (At5g01100), which encodes another member of the putative *O*-fucosyltransferase family, shows a cell adhesion defect at the seedling level that is strikingly similar to that of *quasimodo* (Neumetzler et al., 2012). We tested whether the defects observed in *frb1* and *quasimodo* were genetically related and if *esmd1* could rescue the *frb1* defects. Crosses were made to obtain the double and triple mutants (Fig. 2). The single mutants *qua2-1* and *frb1-2* and the double mutant *qua2-1/frb1-2* showed a clear cell adhesion defect (Fig. 2A). However, the double mutants *qua2-1/esmd1-1* and *frb1-2/esmd1-1* and the triple mutant *qua2-1/frb1-2/esmd1-1* showed a clear rescue of the phenotype (Fig. 2A), indicating that a mutation of *ESMD1* is sufficient to prevent the cell adhesion defect induced by a mutation in *QUA1* (Fig. 1), *QUA2*, *FRB1* and even in the double mutant *qua2/frb1*.

Interestingly, the *qua2-1/frb1-2* double mutant did not seem to show an additive phenotype compared with each single mutant (Fig. 2A). Since a cell adhesion defect is hard to quantify, we looked at the effect of the mutations on dark-grown hypocotyl elongation as a proxy to estimate the strength of the phenotype. *qua2-1* and *frb1-2* hypocotyl elongation is substantially impaired by the loss of cell adhesion, but is rescued by *esmd1-1* (Fig. 2B). Defective hypocotyl elongation in the *qua2-1/frb1-2* double mutant is also rescued by *esmd1-1* and does not show a further reduction compared with each single mutant, supporting absence of additivity in the phenotype.

These results show that *qua2-1* and *frb1-2* are likely to be affected in the same pathway and, overall, demonstrate that mutations in *QUA1*, *QUA2*, *FRB1* and *ESMD1* affect cell adhesion through a common pathway. Furthermore, our results reveal the opposite effects on cell adhesion of mutations in two putative *O*-fucosyltransferases.

### Cell adhesion does not only rely on HG content in the cell wall

**Fig. 3.**
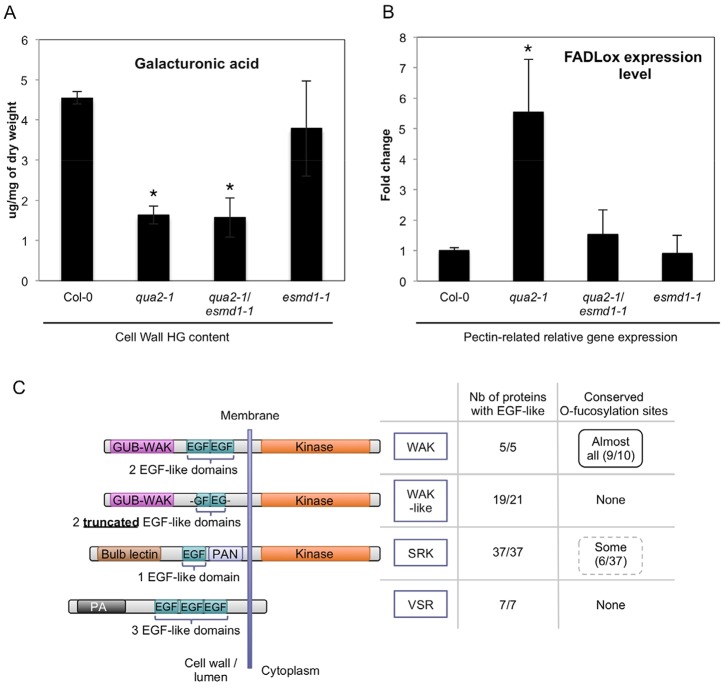
**Cell wall homogalacturonan (HG) content, pectin-related signaling and potential substrates of *O*-fucosyltransferases.** (A) Galacturonic acid content (constitutive monomer of HG) measured on HG-enriched cell wall extracts from Col-0, *qua2-1*, *qua2-1/esmd1-1* and *esmd1-1*. (B) Expression levels of *FADLox* in Col-0, *qua2-1*, *qua2-1/esmd1-1* and *esmd1-1* seedlings. Expression is fold change relative to Col-0. (A,B) Average with s.d. of three biological replicates; *P<0.05 (*t*-test) versus Col-0. (C) Potential substrates of ESMD1 and FRB1 as revealed by UniProtKB/Swiss-Prot database searches. Four classes of proteins contain EGF-like domains: the wall-associated kinases (WAKs); the WAK-like; the S-domain receptor-like kinases (SRKs); and the vacuolar sorting receptors (VSRs). Only the WAKs and some of the SRKs actually have the conserved site for O-fucosylation. GUB-WAK, galacturonic acid binding domain–wall-associated kinase; PA, protease-associated domain; PAN, PAN module. The drawing of each protein type/family is intended to be an average representative structure, but variations exist within families (see Table S1). Not all the proteins considered as part of these families had conserved EGF-like domains (e.g. there are 21 WAK-like proteins, but only 19 have EGF-like domains).

Over recent decades the majority of studies investigating cell adhesion have pointed to a crucial role for HG in cell adhesion (Daher and Braybrook, 2015; Jarvis et al., 2003), including studies of *quasimodo* mutants, in which the loss of cell adhesion was explained as resulting directly from the decreased HG content in their cell walls (Bouton et al., 2002; Mouille et al., 2007). However, this might be a too simplistic view. We aimed to determine if the restoration of cell adhesion in the *qua2-1/esmd1-1* double mutant is accompanied by a restoration of HG content in the cell wall. Galacturonic acid content was measured in an HG-enriched cell wall fraction from 5-day-old dark-grown hypocotyls of the different lines. This revealed that the HG defect of *qua2* was not rescued in the suppressor line (Fig. 3A), indicating that the restoration of cell adhesion by *esmd1* is not due to a restoration of HG content.

To investigate further the causes of the cell adhesion defect and its rescue in the mutant and suppressor lines, we carried out a neutral sugar analysis of the cell wall (Fig. S4). However, no major differences were found. Interestingly, characterization of the *frb1* mutant as reported by Neumetzler et al. (2012) shows cell adhesion defects without a decrease in HG content. Further cell wall and transcriptomic analyses revealed various cell wall modifications potentially responsible for the loss of cell adhesion but the authors could not conclude as to their actual effect. The various cell wall analysis techniques used by Neumetzler et al. (2012) and in our study show that cell adhesion relies on as yet unidentified properties of the cell wall, as a future avenue of research.

### A constitutive pectin-related signaling is associated with the loss of cell adhesion

To determine whether the mutations in *QUASIMODO* and *ESMD1* could affect a pectin-related signaling pathway, we analyzed the expression levels of *FAD-LINKED OXIDOREDUCTASE* (*FADLox*), a gene known to be responsive to pectins (Denoux et al., 2008; Kohorn et al., 2014). The expression level of *FADLox* was ∼5-fold higher in *qua2-1* than in the wild type, *qua2-1/esmd1-1* and *esmd1-1* (Fig. 3B). This suggests that a pectin-related signal is induced in *quasimodo*, and repressed by *esmd1*, which interestingly correlates with the loss and restoration of cell adhesion, respectively.

**Fig. 4.**
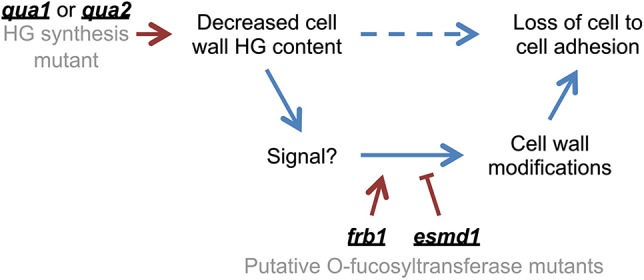
**Model of *qua*, *esmd1* and *frb1* impact on the control of cell adhesion.** Our previous understanding was that the decreased HG content in the cell wall of the *quasimodo* mutants was directly responsible for the loss of cell adhesion. The current work instead shows that *esmd1* restores cell adhesion in the *qua2* mutant without restoring the HG content in the cell wall. We also demonstrate that *esmd1* and *frb1* mutants have opposite effects on cell adhesion that are independent of the HG content in the cell wall. On this basis, we propose that the pectin deficiency in the *quasimodo* mutants is not directly responsible for the loss of cell adhesion (dashed arrow), but instead creates a signal that activates a signaling pathway leading to the loss of cell adhesion. The *frb1* and *esmd1* mutations affect this pathway in a positive and negative manner, respectively, thus triggering or suppressing the cell adhesion defect through as yet unidentified cell wall modifications. The blue arrows represent the previously described (dashed) and newly identified (solid) pathways implicated in cell adhesion. The red arrows illustrate the effect of the mutation on these pathways.

Overall, and as proposed in Fig. 4, our results show that the state of cell adhesion is not a direct structural consequence of the HG content in the cell wall. Based on our results, an alternative hypothesis is that the initial HG decrease in *quasimodo* is perceived, and through signaling leads to more complex cell wall modifications that ultimately cause the loss of cell adhesion. We also uncovered the involvement of putative *O*-fucosyltransferases in cell adhesion by identifying ESMD1 and by involving FRB1 in this pathway. These results modify our understanding of cell adhesion in plants and, interestingly, this situation is reminiscent of recent reports that some of the phenotypes primarily described in cell wall mutants are actually the consequence of cell wall integrity sensing and signaling, leading to disturbed growth and development (Hématy et al., 2007; Wolf et al., 2012a,b, 2014). In addition, knockdown of DEK1, a calcium-dependent protease involved in the signaling leading to epidermal cell differentiation, was shown to affect epidermal cell adhesion (Galletti et al., 2015; Johnson et al., 2005). Along with these previous reports, our work uncovers the idea that a complex signaling pathway is involved in the control and maintenance of cell adhesion in plants.

Future investigations on the function of ESMD1 and FRB1 and the involvement of other cell adhesion defective mutants in this pathway, as well as the characterization of the other suppressors that we have isolated here, will surely clarify and broaden our understanding of the complex mechanisms that control cell adhesion in plants.

## MATERIALS AND METHODS

### Plant material, growth conditions and genotyping

*Arabidopsis thaliana* seedlings were grown at 20°C on solid custom-made medium (Duchefa Biochemie) with various agarose and sucrose concentrations depending on the experiment. Seeds were cold treated for 48 h to synchronize germination, and the plants were grown in a 16 h light/8 h dark cycle. For dark growth conditions, seeds were exposed to light for 4 h to induce germination, then the plates were wrapped in three layers of aluminum foil. Seedling age was counted from the start of light exposure. Genotyping by PCR is described in the supplementary Materials and Methods.

### Mutagenesis, genetic suppressor screen and sequencing

For both *qua1-1* (Bouton et al., 2002) and *qua2-1* (Mouille et al., 2007) ∼15,000 seeds were EMS mutagenized (Kim et al., 2006). M1 plants were bulked in groups of 100-150 and M2 seeds were screened on a low agarose (0.2%) and high sucrose (3%) medium. Suppressor lines were backcrossed twice with either *qua1-1* or *qua2-1*. Illumina sequencing of pools of ∼200 backcrossed F2 suppressors per M2 suppressor line was carried out at The Genome Analysis Centre (TGAC, Norwich, UK). Sequencing data were analyzed using the SHORE (Ossowski et al., 2008) and SHOREmap (Schneeberger et al., 2009) pipelines for the identification of SNPs and indels and their frequency for the bulk segregant analysis. Further details of the genetic mapping, whole-genome sequencing and the identification of ESMD1 as the causal mutation are provided in the supplementary Materials and Methods.

### Protein structural analysis

Potential *O*-fucosyltransferase substrates were identified on the basis of containing EGF-like repeats. Conserved structural and functional domains were identified in ESMD1 by comparison with other *A. thaliana* putative *O*-fucosyltransferases. For details, see the supplementary Materials and Methods.

### Propidium iodide staining and confocal microscopy

Four-day-old dark-grown hypocotyls were immersed in 0.2 mg/ml propidium iodide (Sigma) in water for 10 min and washed with water prior to imaging. Hypocotyls were placed between glass slide and coverslip separated by 400 μm spacers to prevent tissue crushing. Images were collected using a Leica TCS SP8 confocal microscope. Propidium iodide excitation was performed using a 552 nm solid-state laser and fluorescence was detected at 600-650 nm.

### Hypocotyl length measurements

Four-day-old dark-grown hypocotyls were vertically grown and three biological replicates of 15 seedlings each for each mutant were assessed. Images were taken with an image scanner and hypocotyl length was measured using ImageJ (NIH).

### Cell wall composition analyses

Uronic acid content was measured from saponified and ammonium oxalate-extracted pectins as described (Blumenkrantz and Asboe-Hansen, 1973). Neutral monosaccharide composition in cell wall extracts was quantified by chromatography. For experimental detail, see the supplementary Materials and Methods.

### ESMD1 expression constructs

*p35S::ESMD1:GFP* and *pESMD1::uidA* were constructed by Gateway cloning (Invitrogen). The GFP-tagged ESMD1 was assessed following transient expression in *Nicotiana benthamiana*. Expression of the *ESMD1* promoter construct was assessed, following transformation of *A. thaliana*, by GUS staining. For details, see the supplementary Materials and Methods.

### Gene expression quantification

Total RNA was extracted from 8-day-old light-grown seedlings using the RNeasy Plant Mini Kit (Qiagen) according to the manufacturer's instruction and with on-column DNA digestion using RNase-free DNase (Qiagen). *FADLox* (At1g26380) (Denoux et al., 2008) expression level was normalized to that of *GAPDH* (At1g13440) and *UBQ10* (At4g05320) (Czechowski et al., 2005) according to the ΔΔCt method (Livak and Schmittgen, 2001). A detailed procedure is provided in the supplementary Materials and Methods.
